# Cancer and Tumour Suppressor p53 Encounters at the Juncture of Sex Disparity

**DOI:** 10.3389/fgene.2021.632719

**Published:** 2021-02-16

**Authors:** Sue Haupt, Ygal Haupt

**Affiliations:** ^1^Tumor Suppression Laboratory, Peter MacCallum Cancer Centre, Melbourne, VIC, Australia; ^2^Sir Peter MacCallum Department of Oncology, The University of Melbourne, Parkville, VIC, Australia; ^3^Department of Clinical Pathology, University of Melbourne, Parkville, VIC, Australia; ^4^Department of Biochemistry and Molecular Biology, Monash University, Melbourne, VIC, Australia

**Keywords:** p53, sex disparity, cancer, oxidative stress, SNPs, epigenetics, post translational modifications, non-coding RNAs

## Abstract

There are many differences in cancer manifestation between men and women. New understanding of the origin of these point to fundamental distinctions in the genetic code and its demise. Tumour suppressor protein p53 is the chief operating officer of cancer defence and critically acts to safeguard against sustained DNA damaged. P53 cannot be ignored in cancer sex disparity. In this review we discuss the greater prevalence and associated death rates for non-reproductive cancers in males. The major tumour suppressor protein p53, encoded in the *TP53* gene is our chosen context. It is fitting to ask why somatic *TP53* mutation incidence is estimated to be disproportionately higher among males in the population for these types of cancers compared with females? We scrutinised the literature for evidence of predisposing genetic and epigenetic alterations that may explain this sex bias. Our second approach was to explore whether redox activity, either externally imposed or inherent to males and females, may define distinct risks that could contribute to the clear cancer sex disparities.

## Introduction

**FIGURE 1:**
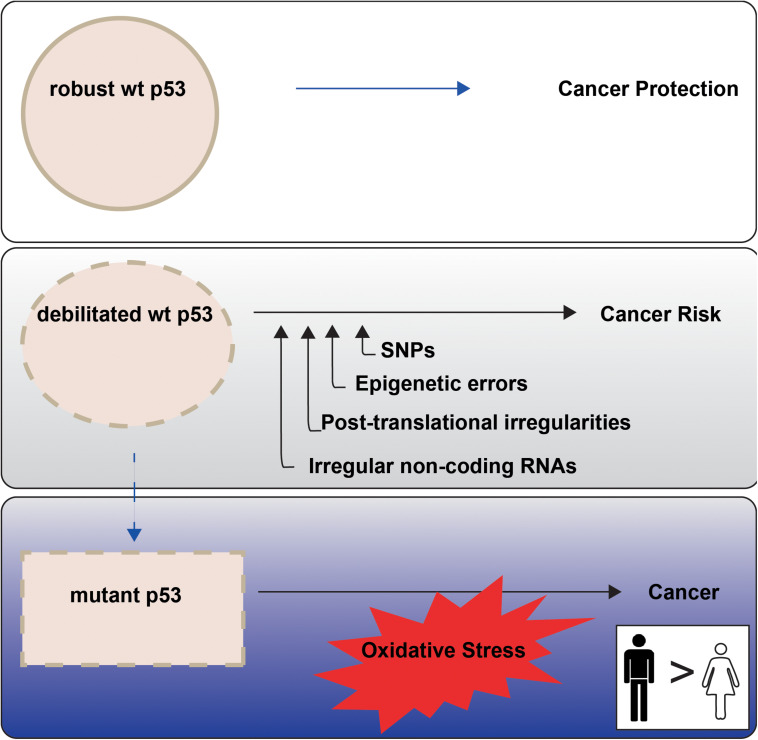
p53 tumour suppressor activities are not equally enforced between the sexes in non-reproductive cancers. P53 is termed the Guardian of the Genome (Lane, 1992) because of its key role in cancer prevention (top panel). P53 function may be less robust when undermined through cell intrinsic events, with emerging sex disparities (middle panel; “Altered TP53 Regulation and Its Cancer Risks for Females and Males” section). *TP53* mutation is a particular risk for non-reproductive cancers in males (blue tonings), with oxidative stress an imposed danger (bottom panel; “Cancer Sex Disparity Linked to Redox Activity Through p53” section).

Higher cancer incidence and mortality in males than females point to sex differences in onset and progression. This is evident among the most common non-reproductive cancers (Cook, 2013). The principal orchestrator of cancer defence in the human body is the tumor suppressor p53, encoded by the *TP53* gene. In response to cellular stress, wild type (wt) p53 protein is amassed, with consequent potentiation of its multiple remedial pathways. The cumulative effect is to protect genomic integrity and prevent the conveyance of genetic damage into future generations of cells (Figure 1, upper panel). The weakening or loss of proper wt p53 control is consequently a serious cancer risk. Indeed, undermining p53 function has emerged as a near universal trait of cancers (Levine, 2020).

In this review we discuss evidence that p53 dysfunction poses distinct risks between the sexes, leading to disparity in cancer outcomes for males and females. We examine sex-specific p53 efficacy and disruption. We explore whether females and males provide a different molecular context to respond to stress conditions and insults. We examine inequities in p53 capacity and consider factors that contribute. At this early stage in the understanding of cancer sex disparity, we point to areas that beg to be researched to inform therapeutic advancement.

## Altered *TP53* Regulation and Its Cancer Risks for Females and Males

Evidence is emerging that certain sex disparities in cancer correspond with different p53 functional capacities between males and females (e.g., Freudenstein et al., 2020). This indicates that p53 cannot perform its activities equivalently between the sexes because of either inherent or externally imposed influences. In a healthy response to cellular stress, p53 is stabilised to increase its operative force to repair and clear up inflicted damage. As a transcription factor, p53 engages specific DNA responsive sequences in the regulatory regions of its target genes and drives their expression (Fischer, 2017). Alternatively, it acts to suppress other sets of genes through the p53-DREAM pathway, involving its key transcriptional target Cyclin dependent Kinase Inhibitor 1A [also known as P21, encoded in *CDKN1A* (Engeland, 2018)]. The level of p53 accumulated and its precise protein folding all critically influence its capacity to effectively activate its response to cellular stresses. Impairment of p53 tumour suppression function is universal in cancers. During cancer development, p53 is either stripped of its power through DNA mutation, as occurs in around 50% of human cancers; or by functional disruption, as implicated in the remainder (Olivier et al., 2010; Donehower et al., 2019). *TP53* is outstanding as the most altered gene in cancer and no other gene approaches its rate of mutation (Soussi and Wiman, 2015).

In this section we introduce a spectrum of mechanisms through which p53 activities are compromised, with associated risks for cancer development in females and males (Figure 1, middle panel). First, we introduce the importance of sex to *TP53* mutation. Second, we examine components of the inherent genetic make-up of healthy individuals that confer distinct cancer defence capabilities. Third, we overview weak or damaged links across p53 pathways that dull stress-induced responses. We identify these features across the genome. Attention is paid to emerging evidence that genetic sex, as normally defined by the sex chromosomes: male XY or female XX, is relevant to p53 functional engagement in cancer defence.

### Disproportionate *TP53* Mutation Incidence Between the Sexes

Our recent work predicts an overall higher incidence of non-reproductive cancers with *TP53* mutation among males in the United States (US) population, compared with females. This statistical assessment was undertaken by drawing on population and cancer databases. Specifically we combined comprehensive quantification of cancer rates in the US population (Surveillance and, Epidemiology and End Results, SEER data), with *TP53* mutation rates in US cancers (as identified in The Cancer Genome Atlas, TCGA data) (Haupt et al., 2019a). The reasons for this disparity remain to be fully identified, but it is pertinent to at least 12 major cancers. An important highlight from this work is that analyses comparing equal numbers of cancers from males and females may define cancer vulnerabilities, but they miss the relative population risk. More explicitly, current cancer data bases do not reflect representative population sampling. Our analyses of *TP53* mutation illustrate the illuminating power of population studies for exposing the breadth of male/female cancer disparities. Further valuable refinements of this approach will include comprehensive comparisons of age groups and population ethnicities.

Examining the impact of *TP53* mutation on overall survival across these 12 prominent non-reproductive cancers together, indicates that *TP53* mutation corresponds with poorer survival (Haupt et al., 2019a). Importantly, we demonstrated that among individual cancer sub-types this may vary, as we recently reported for non-small cell lung cancer. Among lung adenocarcinoma patients, mutant *TP53* cancers correspond to the poorest outcomes, while females with wt p53 have the best overall survival. This is not the case in lung squamous cell cancer however, where *TP53* status overrides sex as a survival discriminator. Unpredictably, the minor group with wt *TP53* in this subtype have better outcomes, predicting a peculiar etiolgy (Freudenstein et al., 2020). These studies emphasise that the impact of sex is not uniform across all cancer types.

What leads to the high incidence of *TP53* alteration in males, associated with sporadic cancers, is the central topic of the following discussion. We now transition to explore factors that can predispose to cancer, in advance of functional disruption of p53 by mutation of its encoding gene, or through alternative surrogate disruptions. We discuss the relevance of the sexes to these topics.

### SNPs in the p53 Pathway and Their Impact on Cancer Sex Disparity

Altering p53 levels or structure jeopardises its associated functions. P53 may be directly or indirectly disrupted. Single Nucleotide Polymorphisms (SNPs) may impact the folding of the translated protein product and also its post-translational modifications (reviewed in Knappskog and Lonning, 2011). SNPs in the p53 pathway affect its performance, in contrast with SNPs in other prominent cancer genes that lack functional impact (Zhao et al., 2018). Knowledge in this area is still growing, despite enormous effort, as we review in the context of the sexes.

#### P53 SNPs as Cancer Risks in Males and Females

The most common and extensively studied p53 SNP dictates the identity of amino acid 72. This SNP encodes a non-conserved change of the wild-type amino acid variants Arginine (CGC) and Proline (CCC) (Arg72Pro–dbSNP ID: rs1042522). These alternative amino acids alter p53 structure, which impacts its performance, with consequence for cancer defence. This SNP resides in exon 4, in the region encoding the p53 proline-rich domain that is fundamental for its regulation and function, including apoptosis (Berger et al., 2005; reviewed by Basu and Murphy, 2016). Evidence that this may not impact equally between the sexes is emerging as we discuss.

P53 SNP72Arg is notorious for its robust apoptosis proficiency (Dumont et al., 2003; Frank et al., 2011). SNP72Pro by contrast, is more efficient at inducing growth arrest and senescence (Bonafe et al., 2003, 2004; Pim and Banks, 2004; Azzam et al., 2011; Frank et al., 2011). The consequence is that SNP72Pro is less capable of eliminating cells with DNA damage, which translates to weaker tumour suppression capability. Coupled with this is a slow self-renewal capacity of stem/progenitor cells. The anathema is that despite associated cancer risk with SNP72Pro, individuals that do not succumb to this have extended longevity (Zhao et al., 2018). This is elegantly demonstrated by the Hu lab in HUPKI mice (human p53 knock-in mice), where mice with p53SNP72Pro developed shorter tumour-associated lifespan, compared with those with SNP72Arg, despite similar tumour incidence. In contrast, mice with p53SNP72Pro that did not perish from cancer, had extended survival than their SNP72Arg counterparts. A rational explanation of these findings is that p53 function teeters on a single nucleotide, either executing a robust apoptotic response to DNA damage, or conferring a life-time delay in replicative exhaustion (Zhao et al., 2018 and references within). Corresponding findings of longevity were evident among certain European and Scandinavian ethnicities (referring to people that are genetically alike through common heritage; Smetannikova et al., 2004; van Heemst et al., 2005; Bojesen and Nordestgaard, 2008). Adding the criteria of sex to the analyses, females from a German cohort have an exclusive increase in longevity associated with SNP72Pro, while males show no association (Gross et al., 2014).

The alternative outcomes of either poorer survival from cancer or healthy extended survival are an unexpected combination that has likely confounded study analyses. An additional source of potential ambiguity is the tissue-specific impact of these SNPs [Azzam et al. (2011) together with others discussed below]. This may be further compounded by distinct SNP prevalence in different ethnicities (reviewed by Basu and Murphy, 2016). For example, p53 SNP72Pro is more frequent in Chinese and African-American patients than Caucasians (reviewed in Olivier et al., 2010). Among Chinese patients with lower rectal cancer, p53 SNP72Pro associates with diminished p53 protein levels. In this cohort, females experienced extended survival compared with males (Zhang et al., 2019). Chinese Han males with this SNP had increased propensity for Non-Hodgkin lymphomas than females (Fan et al., 2014). In contrast, increased cancer risk associated with this SNP in Chinese non-smoking females with small cell lung adenocarcinoma (Ren et al., 2013). These studies highlight the importance of defining genetic context in order to gain a proper understanding of risks across cancer types and between the sexes. These studies demonstrate the danger of drawing simplistic conclusions regarding p53 and sex.

#### MDM2 SNPs in Female Cancer Predisposition

The major negative regulator of p53 is MDM2 (Mouse Double Minute 2 homolog) (Haupt et al., 1997; Kubbutat et al., 1997). In non-stressed cells, MDM2 acts as the major E3 ubiquitin ligase of p53 to keep it restrained and stem its potency. In response to stress, when tumour suppressive p53 activities are rapidly needed, MDM2 constraints are relieved through post-translational modifications. Accumulated p53 in turn feeds back to increase *MDM2* RNA levels, leading to quenching of the unleashed response. Impotence in any step of this tight regulatory cycle poses cancer risk (reviewed in Karni-Schmidt et al., 2016), and sex disparity is emerging in functional competence of this process.

Chronically elevated levels of *MDM2* are oncogenic and pose a threat to p53 anti-cancer function, with serious clinical consequence (reviewed in Karni-Schmidt et al., 2016). Enhanced *MDM2* transcription and protein accumulation from *MDM2* SNP309 (T309G, rs 2279744*)*, is a tangible spontaneous cancer risk due to its capacity to inhibit p53 accumulation in response to stress (Bond et al., 2006). Earlier-age cancer onset (Bond et al., 2006) is associated with this SNP in multiple cancers, with outstanding female disadvantage for colorectal cancer, soft tissue sarcomas, diffuse large B-cell lymphoma (Bond and Levine, 2007) and non-small cell lung cancer (Lind et al., 2006), linked to estrogen signalling (reviewed in Barnoud et al., 2019).

The explanation for increased MDM2 levels is that SNP309 G/G, located in its P2-promoter in its first intron, enhances binding affinity for transcriptional activator steroid receptor transcription factor Sp1, compared to the wt T allele. This chronologically elevates levels of *MDM2* mRNA and protein, which reduces p53 response (e.g., to drug-induced DNA damage caused by etoposide) (Bond et al., 2004). Modelling the *SNP309G* in mice corroborates its correlation with exacerbated tumor burden [as comprehensively reviewed by the Murphy group (Barnoud et al., 2019)]. In keeping with the p53 SNP72, tissue-type dependency and ethnicities are likely to at least partially explain inconsistencies in some study findings regarding the impact of *MDM2* SNP309G.

Drawing upon the example of non-small cell lung cancer specifically, a comprehensive meta-analysis identified *MDM2* SNP309G as a particular female survival risk, but not among all ethnicities. No significant associated risk for this SNP was evident in the Caucasian population, while in contrast, susceptibility was measured in the Asian population and most outstandingly among females (Luan et al., 2019). Consistently, *MDM2* SNP309G is linked to increased Chinese female lung adenocarcinoma risk (Ren et al., 2013). Why the Asian population is particularly susceptible requires further explanation (Hu Z. et al., 2007). These findings await testing on additional populations, which are yet to be adequately sampled, such as those of Africa. Lack of inclusion of comprehensive information regarding patient age, tumour stage, and smoking status, question whether this SNP remains undervalued for its capacity as a biomarker (Luan et al., 2019)?

An addendum to these studies is that in non-small cell lung cancers in female patients from China (Beijing hospital), MDM2 protein levels were identified by immunohistochemistry to be at higher levels both in the cytoplasm and nucleus, compared with lower levels and nuclear location in the cancer adjacent tissues. These measurements were mainly from adenocarcinoma samples and were not apparent in males (Wang et al., 2018). Together these findings expose that males and females are prone to distinct types of risks for non-small cell lung cancer in a cell-type dependent and ethnic-dependent manner, which in turn argues for stratifying patients into sub-groups, with sex-distinct considerations for treatment. In this instance Asian females would be predicted to benefit particularly from MDM2 inhibitors, which are being actively developed for the clinic (Konopleva et al., 2020).

Adding to this, a second P2-promoter SNP, *MDM2* SNP285C, lowers Sp1 binding and essentially counteracts the increased affinity of SP1 for the *MDM2* SNP309G, located 24 base pairs downstream (as reported in female reproductive cancers). This SNP also overlaps an estrogen-receptor binding site of the *MDM2* promoter and is functionally disruptive. This offers explanation for its association with reduced incidence of a number of female reproductive cancers (Knappskog et al., 2012). It also suggests danger for unguided administration of supplementary estrogen post-menopause [reviewed in Grochola et al. (2010)]. Notably, the *MDM2* SNP285C was not found in the Chinese population, in contrast to other ethnicities. This offers a plausible explanation for the selected impact of *MDM2* SNP309G in the Chinese population, in contrast to the ambiguous findings in the Caucasian population (Knappskog and Lonning, 2011). Of relevance, comparable to the weakened p53 response associated with p53 SNP72Pro (above), females with *MDM2* SNP309G who do not succumb to cancer, exclusively enjoy increased longevity. This is consistent with higher MDM2 levels diminishing the potency of the p53 stress response yet extending the duration of stem cell repopulation among survivors. This correspondence was measured among female centenarians who were heterozygous or homozygous for this allele, while males showed no association (Gross et al., 2014). These findings are relevant to sex disparity within individual populations but argue that overall higher male cancer incidence must be associated with other causes.

### Epigenetic Dysregulation of *TP53* and Its Pathways Between the Sexes

Direct incapacitation of p53 is a clear risk for sustaining genetic damage. In addition, sabotaging the cancer defence barricades normally enlisted by p53 is a significant danger. In this context, preventing the production of barrier components is a peril. An example is interference with the production of vital protein defence molecules, by halting transcription of their DNA into RNA, which in turn curtails translation into protein. Methylation of gene promoters is an enzymatic modification that is installed post-replication and typically modifies the cytosine in CpG dinucleotides and is associated with histone acetylation (Ehrlich, 2009 and references within). It is relevant to acknowledge that DNA methylation patterns differ between healthy males and females [e.g., in skeletal muscle (Davegardh et al., 2019)] suggesting that cancers develop onto this dimorphic background. Methylation of gene promoters silences the output of their encoded protein products. Therefore, hypermethylation of gene promoters can pose an alternative cancer risk to gene mutation. Specifically, hypermethylation of the promoters of tumour suppressors may prime unregulated proliferative growth.

Importantly, sex disparity has been linked to methylation of genes encoding p53 pathway components. Gastric cancer (stage II and III) illustrates the danger of elevated methylation of genes in the p53 pathway. In a wt *TP53* context, greater male risk is linked to three genes in the p53 pathway that are disrupted by methylation. Females on the other hand have lower rates of methylation and have lower gastric cancer risk. The genes in primary tumours with extremely high levels of methylated promoters in these cancers are *PGP9.5, CCNA1*, and *NMDAR2B*. These are proposed tumour suppressor genes: with PGP9.5 able to bind and stabilise p53 by preventing its proteasomal degradation; and NMDAR2B and CCNA1 acting to facilitate apoptosis (Waraya et al., 2015). The therapeutic importance of these findings is that epigenetic modifiers able to reduce methylation [e.g., 5-aza-2’-deoxycytidine (5-aza-dC), a DNA methyltransferase 1 (DNMT1) inhibitor]; in combination with sodium valproate or valproic acid [a histone deacetylase inhibitor (Rocha et al., 2019)], will have specific value, with greater relevant application in these male gastric cancers, due to their higher methylation incidence. Altered methylation (either 11 hyper-methylated and seven hypomethylated) of ∼30% of genes (18/67) in the p53 KEGG pathway were also found in colorectal cancer (Molnar et al., 2018), which now begs for interrogation of sex differences. These intriguing findings emphasise the emerging role of epigenetic deregulation in cancer, which demands further examination with respect to patient sex. Unrecognised methylation phenomena are expected to confound the interpretation of SNP studies in cancer.

### Post-translational Modifications of p53 Dictate Its Fate

Cellular levels of p53 are dynamically regulated at the protein level predominantly, through post translational modifications, rather than by transcriptional control (Donehower et al., 2019). As discussed above, MDM2 is the key E3 ligase governing p53 protein levels. P53 basal levels are kept low under normal conditions, but in response to stress, MDM2 releases p53 through rapidly installed, finely choreographed responses. This aligns with the importance of MDM2 levels, as relevant to its SNPs as discussed above. Inherent in the activation of p53 is its phosphorylation at Ser-15, Ser20, Thr18, and Ser-46. Important upstream activators of p53 are ATM (encoded by *Ataxia-telangiectasia kinase* gene), ATR and CHEK2 (checkpoint kinase 2). Through a phosphorylation cascade, these kinases regulate p53 at its protein level. ATM activates CHEK2 which in turn inhibits MDM2 with consequent relief of p53. CHEK2 also phosphorylates p53 leading to upregulation of cell cycle inhibitor p21. Glycosylation coordinates with phosphorylation in p53 regulation, and its role appears to be vital to cellular function, well beyond a mere place-holder role (Hart et al., 2011). How sex impacts these processes is relevant to cancer.

#### Kinase Modifications of p53 and the Sexes

Discriminating sex-disparity in post-translational p53 modifications relies on proper subject matching for robust comparisons. This is challenging in humans, where potential inequity may arise from inherent SNPs (e.g., as discussed with p53 SNP72; or across its pathways). The value of inbred animals becomes obvious in this light, as they offer a system for evaluating effects of individual molecular alterations between the sexes. However, they have emerged with their own concerns. To illustrate, we discuss data from mouse models in which sex disparity in p53 up-stream regulators ATM and CHEK2 is observed (where we use the human HUGO terms for convenience).

Early work from the Levine lab explored the impact of age on p53 function in C57Black6, DBA2, and Balb/c mice and identified that p53 accumulation and transcriptional activity in response to stresses, notably γ-irradiation (IR) declines with age, in a sex-dependent manner. Corresponding with age, there were reductions in levels of the key molecular transponder ATM, which links IR to p53 activation. ATM kinase phosphorylates MDM2 at Ser-395 and p53 at Ser-15. Aging was correlated with reduced responses to other carcinogenic stresses, albeit at a later age than observed in response to IR. In a surprising contrast to humans, males outlived females in this mouse model. Consistently, p53 transcriptional activity declined more slowly with age in males compared with females (Feng et al., 2007). This important study uncovers a critical limitation of these commonly used mice genotypes for pre-clinical modelling of human aging. The relevance of other inbred mouse strains have indeed been questioned with the finding of remarkable dissimilarities in longevity depending on the facility (reviewed in Austad and Fischer, 2016).

This is similarly relevant to studies of mice engineered to model a human truncation variant of CHEK2, which is kinase deficient. Once again, females prove to be significantly more tumour prone with reduced survival, compared with males. Tumours arise predominantly in mesenchymal tissues, the hematopoietic system and lung, with risk also in breast epithelial tissues (Bahassi el et al., 2009). In this latter instance however, germline CHEK2 mutations are a demonstrated risk for human females for breast cancer, due to a suggested link to estrogen. Lung tissue has compensatory mechanisms for CHEK2 inactivity, indicating tissue specific risks that were not evident in the mice (van Jaarsveld et al., 2020). These studies expose the danger of overinterpreting animal models in an attempt to understand the influence of sex on cancer outcomes in humans.

#### Sex Differences in p53 Glycosylation

The addition of *O*-GlcNAc (*O*-linked N-Acetylglucosamine) to p53 at Ser-149 has been linked to stabilising p53 (Yang et al., 2006). X-linked *O*-GlcNAc Transferase (OGT), dynamically modifies p53 introducing a glycosylation event that has been linked to reduced proteasomal degradation of p53 (Yang et al., 2006). This is particularly relevant as *OGT* is an X-linked gene. There are two copies of *OGT* in females and only one in males. Early in embryogenesis, from early trophoblast stage prior to implantation, levels of *OGT* are higher in females, which appears to endow them with greater resilience to prenatal hypothalamic stress compared with males (Nugent et al., 2018).

At an important post-implantation stage, one of the two female X chromosomes becomes silenced and remains so through life. This X chromosome Inactivation (XCI) event occurs to one of the female X chromosome pair randomly. This silencing is executed by the lncRNA (long non-coding RNA) *XIST* (*X-inactive-specific transcript*). While vital for initiation of XCI (as discussed in greater detail below), maintenance of this chromosome silencing appears to involve methylation (with further discussion of these elements in the following sections). This process is necessary for equating gene expression levels of *OGT*, and most other X-linked genes between the sexes (Barros de Andrade et al., 2019).

An intriguing clinical finding relates to the application of chemotherapy 5-azacytidine and OGT levels exclusively in females. Even though *OGT* undergoes XCI in females, in response to treatment with this agent, its expression at least doubles in female human fibroblasts, without impact in males. This drug blocks DNA methylation, which likely then disrupts its capacity to maintain methylation of the inactivated copy of *OGT*. This provides a rational explanation for its particular response elicitation in females (Olivier-Van Stichelen and Hanover, 2014). Further exploration of the impact of cytidine-based drugs between the sexes is warranted as differences are emerging in their application in cancer (e.g., Di Florio et al., 2020). Tissue and mutational context are relevant parameters to consider also in this type of therapeutic approach (e.g., Luanpitpong et al., 2017).

### Non-coding RNAs in the p53 Pathway and Cancer Sex-Disparity

Considerations of non-coding RNA (ncRNA) in cancer sex disparity and its links to the clinic are only recently emerging. This is remarkable given the prevalence of >50,000 ncRNAs. These are transcribed from the non-protein coding component of the genome, which constitutes ∼98% of its entirety (Slack and Chinnaiyan, 2019). The most studied ncRNAs are microRNAs (miRNAs; ∼22 nucleotides; reviewed in Rupaimoole and Slack, 2017) and long non-coding RNAs (lnRNAs; >200 nucleotides), which include the subclasses of pseudogenes and circular RNAs. While miRNAs target RNA, lncRNA may functionally engage DNA (e.g., *XIST*), miRNAs or proteins (reviewed in Slack and Chinnaiyan, 2019). NcRNAs are underexplored in cancer sex disparity but some vital links to p53 have emerged.

#### The lncRNA XIST—p53 Axis in Females

Deregulated ncRNAs are a tangible cancer risk for p53 itself and also its negative regulators. With our colleagues we uncovered a fundamental connection in females between lncRNA *XIST* and p53. We discovered that p53 controls X chromosome silencing by up-regulating *XIST* levels in mice (Delbridge et al., 2019). This is a female-specific regulation. Loss of p53 reduces *XIST* expression, which in turn derails the fundamental XCI event and drives embryonic lethality in female mice. It is important to reiterate that normal healthy females carry an XX chromosome genotype but can only tolerate the full expression of a single X chromosome. XCI halts the expression of ∼85% the X-encoded genes on one of the two female X chromosomes (XX), which limits gene expression to a level similar to that of XY males. The set of X chromosome genes that are not silenced are referred to as XCI escaper genes.

A very important set of studies from the Lee lab demonstrate the danger of *XIST* depletion for female cancer. Unexpectedly, mice with post-implantation *XIST* deletion are generally remarkably tolerant of this genetic alteration. However, in two instances *XIST* deletion is seen to provoke cancer. Firstly, *XIST* deletion from hematopoietic progenitors results in aberrant proliferative growth, consistent with myelodysplasia [now recognised as cancer (Yildirim et al., 2013)]. Secondly, when *XIST* is selectively deleted from the gut epithelium and carcinogenic stimuli administered, cancer develops (Yang et al., 2020). Of note, both these cellular contexts are highly proliferative, in keeping with more rapid rates of cell division offering greater opportunity for cancer related errors.

On another level, *XIST* is regulated by the vital X-linked epigenetic modifier ATRX. ATRX binding to *XIST* is essential for the recruitment of the polycomb repressive complex 2 (PRC2; Sarma et al., 2014), which in turn contributes to early gene repression events in XCI (Bousard et al., 2019). Such tumour suppressive functions of ATRX are relevant to its mutation/loss across many cancer types (Ren et al., 2020). What is of particular relevance to cancer sex disparity, is our finding that ATRX is among a set of X-linked genes connected to p53 functionally. These genes are preferentially protected from mutant expression in female cancer patients, but not male (Haupt et al., 2019a).

#### X-Chromosome MicroRNAs and the p53 Pathway in Cancer

MiRNAs repress post-transcriptional gene expression and/or degrade messenger RNA in around 30–50% of all protein coding genes across the genome (reviewed in Pinheiro et al., 2011). Each miRNA may have tens to hundreds of predicted mRNA targets, with an average of 90 potential targets. However, engagement does not always result in repression of target gene expression (Liu and Wang, 2019), emphasising the importance of validation for defining function in a cancer and sex context-dependent manner.

MiRNA perturbation is a cancer risk, due to the breadth of their involvement in fundamental cellular and biological processes. Notably, the X chromosome is particularly enriched with miRNAs directed to the “p53 signalling pathway” (Di Palo et al., 2020). MiRNA alterations are widely evident in tumours (Rupaimoole and Slack, 2017). Yet, only very few cancer-specific miRNAs are identified as oppositely expressed between males and females in TCGA (Yuan et al., 2016), with even fewer of their mRNA targets unequivocally identified in cancer tissues (e.g., Guo et al., 2017).

The X chromosome has the highest density of miRNAs in the genome (Guo et al., 2009), comprising ∼10% of all miRNAs. Of the 118 miRNAs detected, 62 have been experimentally validated to date (Di Palo et al., 2020). By contrast, to the X chromosome, the Y chromosome has only four putative miRNAs identified, two are paired with X chromosome miRNAs (reviewed in Di Palo et al., 2020).

Among 106 XCI escaper genes, 6 contain 12 miRNAs in their chromosome loci (Matarrese et al., 2019), but female expression from both chromosomes (or biallelic expression) of these miRNA is largely speculative (reviewed in Care et al., 2018; Pinheiro et al., 2011). Of particular interest to this review is the X-linked miR-504 that targets *TP53* 3’-UTR. Over expression of this miRNA is a cancer risk because it abrogates p53-mediated tumour suppression (Hu et al., 2010). Evidence of sex disparity in this miRNA begs to be tested. Surprisingly, greater differences are identified in miRNAs from the non-sex chromosomes (autosomes), than the sex chromosomes (the autosomes), at least in healthy individuals (Cui et al., 2018), but the relevance of this to cancer predisposition between the sexes also awaits investigation.

One indirect link to p53 and sex disparity is evident in colorectal cancer, through miR-34a whose levels correlate with expression of circadian rhythm clock-gene, PERIOD2 (*PER2)* mRNA (Hasakova et al., 2019). Males with high miR-34a expression have extended survival, but only for early disease stage lacking metastases or node involvement. Importantly, this correlation is lost in males with more advanced disease and does not exist at all in females. In females on the other hand, extended progression-free survival for metastatic colorectal cancer correlates with high levels of miR-192, miR-206, miR-194, and miR-219, with apparent links to proper control of the expression of circadian regulators: *PERIOD*, *CLOCK, BMAL1*, and *CRY*. Comparatively, males with high expression levels of these particular miRNAs have worse outcomes (Garufi et al., 2016). These examples illustrate sex-specific prognostic potential, and add an additional layer to sex-specific links evident through Per2/PML/mutant *TP53*, identified in early mouse models of cancer sex disparity (Gu et al., 2012; Haupt et al., 2013).

MiRNA mimics are in phase I clinical trials alone or together with IR. Their capacity to potentiate immunotherapy by depleting check point molecules, such as PDL1, to relieve T cell exhaustion (Cortez et al., 2016) is underexplored, particularly between male and female cancers. For example, it is rationale to test for sex disparity in the influence of putative PDL1-repressors encoded on the X chromosome: miR-513 (Dragomir et al., 2018), miR-20a/b, miR-106a/b, and miR-424 (Care et al., 2018). Similarly, opportunities to utilise lncRNAs as biomarkers, vehicles for delivery of therapy and also as therapeutic targets (reviewed in Slack and Chinnaiyan, 2019), in a p53-dependent, or sex-specific manner in cancer remain to be extensively studied.

Numerous challenges are associated with determining the role of ncRNAs in cancer sex disparity in general, and more specifically as they relate to p53. Notably, age may influence ncRNA sex disparity (Meder et al., 2014). Tissue, cell or even organelle specificity may be crucial to proper evaluation of miRNA impact between the sexes in cancer (Guo et al., 2017). This is relevant to tumour suppressor or oncogenic function of ncRNAs, where lncRNAs for example have context dependency (Slack and Chinnaiyan, 2019), cautioning against generalisation across cancer types and the sexes.

Human primary cells have good correlation between pre-miRNA and mature miRNAs, rationalising the use of the former as a proxy for the latter (de Rie et al., 2017). It is reasonable to question whether this holds across cancer types and in a sex-dependent manner, where expression is available in TCGA at the level of pre-miRNAs, rather than mature miRNAs (Cui et al., 2018). Currently only a few examples of individual miRNAs that escape XCI are available, despite the production of a miRNA atlas (de Rie et al., 2017). Exact identification of XCI escapers or those with mosaic expression are predicted to be invaluable for cancer sex disparity studies and therapy going forward (reviewed in Care et al., 2018). Longitudinal studies are needed to correlate ncRNA and target mRNA/protein/miRNA levels and these are currently lacking. Further, only limited numbers of datasets exist with normal samples and definitive sex identification [e.g., Gene Expression Omnibus (GEO) database]. MiRNA expression in TCGA tumour-adjacent tissues deviates from normal healthy tissues, calling for caution against over interpretation (Cui et al., 2018). Additional limitations of databases maybe that sex is not assigned in samples (Pinheiro et al., 2011) and that male/female sample numbers are imbalanced (Cui et al., 2018). Epigenetic modifications of ncRNAs is newly recognised in cancer. These are yet to be explored for scope as biomarkers and as therapeutic targets (Esteller and Pandolfi, 2017; Chellini et al., 2020) relevant to cancer sex disparity.

## Cancer Sex Disparity Linked to Redox Activity Through P53

The peculiar vulnerability of males to non-reproductive cancers predicts fundamental inequities between the sexes and raises questions regarding its basis. Firstly, whether males are more inherently exposed to damaging carcinogenic stimuli than females; and secondly, whether males are less able to mount remedial responses to both external and internal genomic damage than females (a concept introduced in “Altered TP53 Regulation and Its Cancer Risks for Females and Males” section)? These are not mutually exclusive, and either or both of these options may be influential as we discuss below. While lifestyle habits such as smoking, alcohol consumption, and workplace risks (related to carcinogen exposure) may traditionally have been higher in men, there are additional inherent influences that appear to disadvantage males (Oliva et al., 2020).

In the following sections we examine sex differences in redox biology that pose greater risks for p53 disruption in males, particularly the mutation of *TP53*. It is not our intention to present a comprehensive review of redox biology in cancer (e.g., Wang et al., 2019) or its intricate links to p53 [as thoroughly done by others, e.g., Liu et al. (2019)]; but rather to extract vignettes pertinent to p53 and cancer-sex disparity. It is relevant to keep in mind that sex hormones are at play, influencing the context into which these oxidative stresses are acting, but it is also important to note that hormone levels decrease with age. The impact of sex hormones on cancer have been extensively reviewed for their distinct effects on cancers in men and women (e.g., Clocchiatti et al., 2016), so we have opted rather to mention them only as specifically pertinent to our chosen focus on redox biology engaging with p53.

Male cancers have greater overall DNA mutation, measured as single nucleotide variants in a pan-cancer study examining both coding and non-coding sequence, at least in non-sex chromosomes (Li et al., 2020). An important parallel is that cancers with *TP53* mutations are also outstanding for their level of chromosomal disruptions (Donehower et al., 2019). This raises further questions concerning why males are at particular risk from non-reproductive cancers: with *TP53* mutation specifically (Haupt et al., 2019a); and also with genome-wide DNA mutation (Li et al., 2020). Presumably this cannot be a mere coincidence? It is also worth noting the exceptional instance of kidney cancer, where sex disparity frequently develops in the context of elevated MDM2 levels, but rarely in the context of *TP53* mutation. This argues that the disruption of the p53 pathway is important in male sex-disparity for these non-reproductive cancers but is also dictated by tissue type-specificity. The example of kidney cancer prevents trivialisation of a correlation between overall DNA mutation incidence and *TP53* mutation (Haupt et al., 2019a). Adding to this is the question of why males accumulate DNA damage at earlier age than females (Podolskiy et al., 2016)?

### Sex Differences in the p53—ROS Axis

Healthy metabolically active human cells generate reactive oxygen species (ROS), which are normally countered within the same cell by neutralising antioxidant pathways. Reactive Nitrogen species are also generated during cellular metabolism (reviewed in Lefaki et al., 2017), however we will focus on ROS particularly as more relevant detail is available on the topic. Measuring resting metabolic energy rates between the sexes has been clouded by whether they should be quantified relative to whole body mass or only the lean component. The lean component, rather than the fat, is argued to be most relevant to routine energy consumption. As females have a higher fat content than males [with healthy body fat estimates of ∼20% for males and ∼30% for females (St-Onge, 2010)], this confuses functional comparisons. When only lean body mass is considered, the resting metabolic rate is lower in females than males (Drolz et al., 2014).

ROS are generated by mitochondria. They originate from mitochondrial oxidative phosphorylation during respiration (Murphy, 2009). Oxidative stress results when ROS levels are not adequately controlled (Lushchak, 2014). Excess ROS can induce DNA damage and lipid peroxidation and in turn disease, including cancer (reviewed in Eriksson et al., 2019). Relevant to the management of ROS, estrogens up-regulate mitochondrial antioxidants, allowing a balanced redox equilibrium that is a female advantage (reviewed in Straface et al., 2017). This predicts that females will have greater capacity to respond to oxidative stress if ROS levels are perturbed from their equilibrium state. Tighter regulation of redox balance in females is predicted to extend their longevity (Austad and Fischer, 2016). This then provides context to the poor capacity of males to manage oxidative stress under physiological conditions (Kander et al., 2017), relevant to their greater inherent cancer risk than females.

Wt p53 and mitochondria are fundamentally engaged in energy production and stress response, as reviewed in depth (Budanov, 2014; Eriksson et al., 2019). We will restrict our discussion to facets relevant to cancer predisposition from the perspective of sex differences. We will keep the concepts at high level with selected specific examples of genes in context, to exemplify linkage to sex. It is important to recognise that *TP53* mutation is likely to perturb these normally highly regulated connections. Throughout these discussions, it is also relevant to keep in mind that given the higher incidence of *TP53* mutations among males for non-reproductive cancers, breakdown of these systems is expected to be more prevalent in males than females.

### P53 Links to ROS Generation and Eradication

Wt p53 promotes mitochondrial biogenesis, through both its transcription factor transactivation function and also its exonuclease activity (references in Beyfuss and Hood, 2018). Consistently, mitochondria are depleted in the absence of wt p53 (Lebedeva et al., 2009). How mutant p53 impacts the mitochondria is an important open question that is relevant to cancer and sex disparity.

ROS are generated by mitochondria during their generation of cellular energy as ATP (adenosine triphosphate), but also as they mediate cell death pathways. Both these functions link to p53. Normal wt p53 regulates respiration through transcriptional transactivation of its targets, but also regulates metabolism through its physical engagement and functional modulation of mitochondrial components *in situ* (reviewed in Budanov, 2014; Liu et al., 2019; as we discuss in more detail in the next section in the context of its relevance to cancer sex-disparity). Further, wt p53 suppresses the mutation of mitochondrial DNA (Lebedeva et al., 2009). This occurs in these maternally inherited organelles with their own DNA. This adds p53-dimension to the concept that mitochondria are better adapted to female context above male, due to their exclusively maternal origin (reviewed in Beekman et al., 2014).

High ROS levels drive insults to induce cell death, involving pro-oxidant induction, while its low levels are linked to resolving modest stress insults consistent with an anti-oxidant function. More specifically, acute, high levels of ROS induce oxidative stress, able to trigger signalling pathways to precipitate cell death, in response to infection for example (reviewed in Di Florio et al., 2020). Similar response is triggered following genotoxic damage, including radiation therapy and chemotherapy, where toxicity is greater in females (Ozdemir et al., 2018).

P53 is tightly linked to redox responses (reviewed in Budanov, 2014; Eriksson et al., 2019). High levels of ROS promote p53 accumulation. Acute ROS signalling is integral to p53 activation pathways that contribute to programmed cell death (apoptosis) or ferroptosis (reviewed in Perillo et al., 2020). Sub-lethal doses of ROS can prime wt p53 to activate repair pathways that are implemented over a time of temporary cell cycle arrest. Sustained ROS levels at sub-lethal doses however are a risk for DNA mutation including *TP53* mutation and in turn cancer development (Figure 1, lower panel). Examples include exposure to cigarette smoke, which is associated with damaging inflammation to the airways (reviewed in Lefaki et al., 2017); and unresolved infections. A relevant example is the higher male than female infection incidence for *Helicobacter pylori*, which can drive stomach cancer (de Martel et al., 2020). The influence of p53 in immunity is an extensive topic (reviewed in Agupitan et al., 2020) with evident sex-disparity [as we identified in non-small cell lung cancers to be linked to *TP53* status and sex (Freudenstein et al., 2020)]. However, other than mentioning its links to infection control through inflammation, we will consider it beyond the scope of this review.

To summarise, this argues that the associated risk of ROS generation from more metabolically active males, even when resting (Drolz et al., 2014), together with enhanced exposure from external sources [e.g., infections (reviewed in Lefaki et al. (2017))], poses a greater risk for cellular redox imbalance than in females. In turn this is more likely to deregulate p53 damage responses and drive male cancers. It is relevant to add that p53 is capable of eliciting targets that activate pro-oxidant activities, but also anti-oxidant functions (reviewed in Budanov, 2014). This apparent ambiguity is becoming better understood with increased understanding of redox biology. It is now clear that oxidants are relevant to control cancer and anti-oxidants are important to preventing uncontrollable oxidative stress accumulation, but their ill-considered administration may also benefit cancer cells. This is seen where antioxidants subdue ROS generation and consequently p53 activation, leading to lung cancer proliferation (Sayin et al., 2014). It is also relevant to note that evidence supports the relevance of ROS levels to chemotherapeutic efficiency with drugs such as cisplatin, where resistance is linked to higher ROS levels pre-treatment (Zaidieh et al., 2019). The impact of ROS begs to be explored in a sex-specific context with *TP53* mutation considerations.

### P53 Links to Sex-Disparity of Cellular Energy Production

A major source of cellular energy is glucose intake. Once in circulation it is transported into cells where it is processed into a functional resource. In normal, healthy cells glucose is processed through the glycolysis pathway to its intermediate pyruvate. It enters the mitochondria and is metabolised progressively through the TCA cycle (Tricarboxylic Acid cycle) and along the oxidative phosphorylation pathway; resulting in the efficient generation of ∼32 ATPs (Melkonian and Schury, 2020). Oxidative phosphorylation may leak ROS that risks health if poorly managed (Nolfi-Donegan et al., 2020). This highlights the relevance of the vital intracellular ROS detoxifier NADPH, which is also produced across these pathways (Mele et al., 2018).

Key enzymes in the glucose metabolic pathways are linked to wt p53 activity. Greater basal male energy demand is consistent with potential for early failure in the maintenance of the antioxidant systems, that then becomes a cancer risk. *TP53* mutation is one direct way to derail the controlled production of cellular energy.

Males are noted for higher levels of circulating glucose (Keramida and Peters, 2017). They also have elevated levels of the main glucose cellular transported GLUT1 (SLC2A1), as measured in lung cancer (Tan et al., 2017), predicting greater capacity for cellular uptake than in females. GLUT1 and its activity induced counterpart GLUT4 are subject to wt p53 regulation (Schwartzenberg-Bar-Yoseph et al., 2004). If p53 becomes mutated, GLUT1 cell surface levels are elevated (Zhang et al., 2013), facilitating increased glucose intake, and reinforcing a sinister feedback loop of mutant p53 stabilisation (Rodriguez et al., 2012).

Additional enzymes regulating glycolysis are also subject to p53 control (Liu et al., 2019). An example is p53-induced glycolysis and apoptosis regulator (TIGAR, encoded in *C12orf5*), that is transcriptionally regulated by wt p53. *TIGAR* loss increases the ability of mutant *Kras*-driven mouse pancreatic cancer to metastasise, particularly in the context of compromised p53 (Cheung et al., 2020). This is relevant to human pancreatic adenocarcinoma, which is around 30% more common and deadly in males (National Cancer Institute, 2020); associated with more frequent *TP53* mutation, than age-matched females (Haupt et al., 2019a).

Another specific example encoded on the X chromosome is Glucose-6-Phosphate Dehydrogenase (G6PD; Jiang et al., 2011) that forms the rate-limiting branch point off the glycolytic pathway onto the alternate pentose phosphate pathway. This pathway is a source of detoxifying NADPH, plus macromolecular building blocks. G6PD is physically inhibited by accumulated wt p53, predicting ROS build-up with potential feed-back to further activate p53 to promote eradication of a damaged cell. Elevated G6PD enzyme activity occurs in esophageal cancer (Wang et al., 2016), a disease with high *TP53* mutation levels (Haupt et al., 2019a), which is ∼4.5 times more frequent and deadly in males than in females (National Cancer Institute, 2020). Inhibition of G6PD is a relevant approach to cancer regulation, and a target of a resveratrol derivative (Mele et al., 2018). Other genes with links to p53 in the oxidation phosphorylation pathway [e.g., SCO2, Synthesis of cytochrome C oxidase 2; GLS2, Glutaminase2 (Budanov, 2014)], will also be important to examine for sex-disparity.

An relevant angle on this topic aligns with greater male frequency of brain tumours called astrocytomas, a type of glioma. We discuss this cancer here as it links metabolism, the X-linked chromosome, and p53 in cancer sex disparity. In this most common type of brain tumour in adults and children, there is functional disruption of three genes sequentially. First isocitrate dehydrogenase (IDH), a key enzyme in the TCA cycle is mutated; followed by p53 loss; then X-linked *ATRX* mutation (introduced earlier). The order of mutational events is crucial to the development of this disease. Mechanistically, IDH mutation causes production of oncometabolite 2-hydroxyglutarate, which competitively inhibits alpha ketoglutarate-dependent dioxygenases to interrupt DNA and histone demethylation cellular activity. Consequent hypermethylation is a risk for epigenetic deregulation of tumour suppressors (Modrek et al., 2017) (relevant to earlier discussion). These examples demonstrate tight links between p53 and metabolism, with emerging relevance to cancer sex disparity.

### P53 Promotes Antioxidants and ROS

Wt p53 contributes to the maintenance of cellular redox balance through its transactivation of key antioxidant targets. Reducing oxidative stress is a valuable tumour suppressive function of p53 and we will briefly mention some of its key targets. Examples of enzyme targets of wt p53 that neutralise ROS include: superoxide dismutase that catalyses superoxide ions to hydrogen peroxide, and Glutathione peroxidase 1 (encoded by *GPX1*) and catalase, which in turn breakdown hydrogen peroxide to water and oxygen (reviewed in Cordani et al., 2020).

Only limited literature appears on the relevance of the sexes to p53 and its impact on antioxidants. One study on rat cortex however, measured higher GXP1 and glutathione reductase levels in brains of older female rats, compared with their male counterparts. Superoxide is also generally higher in the female cortex. On the other hand, in this tissue, increased expression of p53 and p21 is evidently greater in males than females (Tarry-Adkins et al., 2006). Whether this is indicative of other tissues, or ages and how it correlates with humans remains to be tested.

Acute stress, including ROS activation of wt p53 in a seriously damaged cell, invokes powerful responses to ensure its elimination. Apoptosis induction involves p53 transcriptional activation of key targets, but also its translocation to the mitochondria. P53 is able to directly activate BAX to induce mitochondrial membrane disruption and its loss of membrane potential and death (Chipuk et al., 2004). An intriguing link to sex disparity is that BAX elimination from defined regions of the mouse forebrain (e.g., preoptic area) neutralises differences in neuron number that are attributed to greater apoptosis in females (Jyotika et al., 2007). In future studies it will be invaluable to test for sex disparity in the plethora of p53 targets that mediate its apoptotic, ferroptotic, and senescent functions (reviewed in Gnanapradeepan et al., 2018).

### P53 and the Proteasome

Keeping cellular redox in balance is a vital function of the proteasome. It acts by removing and degrading oxidised and damaged proteins and peptides. As a logical corollary, proteasomal inhibition results in increased ROS levels (reviewed in Lefaki et al., 2017), which is a danger for cancer development, but also holds scope for emerging cancer therapies (Manasanch and Orlowski, 2017).

Extensive sexual dimorphism has emerged in proteasome function in mice, with overall higher activity in female intestine, spleen and kidney. Links to longevity are suggested, with females potentially enduring less exposure to damaged cellular peptides and proteins (Jenkins et al., 2020). The function of the proteasome declines with age, although not uniformly, across tissues or between the sexes (Jenkins et al., 2020), with concomitant increase in ROS levels (reviewed in Lefaki et al., 2017) posing increased cancer risk.

P53 and the proteasome engage at many levels. P53 is itself subject to degradation through the proteasome. Entrance into proteasomes is on a ubiquitin-passcode basis and this assignment is normally strictly regulated by a hierarchy of ubiquitin ligase enzymes. The major E3 ligase of p53 is MDM2, which functions in the context of its partner MDM4 (reviewed in Haupt et al., 2019b). Overexpression of these proteins is oncogenic and a risk for cancer, as relevant to our earlier discussion of SNPs, and MDM2 amplification and their examination in the context of sex is warranted. A number of other E3 ligases in specific contexts are also able to target p53, with potential cancer risk if overexpressed (reviewed in Pant and Lozano, 2014). During infection by Human Papilloma Virus (HPV), p53 is targeted to proteasomal degradation through viral E6 protein and the host E6AP (E6-Associated Protein) E3 ligase. It is worth noting that females have greater overall viral resistance, but context is important [particularly as relevant to HPV which is predominantly sexually transmitted; reviewed in Bandilovska et al. (2019)].

At this point it is also pertinent to introduce the elegant discovery from the Del Sal lab that mutant p53 activates transcription of 37 proteasome genes in breast cancer lines. Mutant p53 achieves this by engaging transcription factor NRF2, that is a master regulator of cellular anti-oxidant responses, although one that reduces with age [as reviewed in Lefaki et al. (2017)]. This in turn is a danger for resisting remedial proteasome inhibition. This new function is likely to have been selected among p53 mutants to preserve cellular function in the context of high levels of oxidised and damaged proteins and peptides. Of note, wt p53 is not capable of interacting with NRF2 (Walerych et al., 2016). An unexpected twist is that one of these mutant p53-NRF2 targets is the proteasome subunit PSMD10, which facilitates Mdm2-mediated degradation of wt p53 (Higashitsuji et al., 2005). Whether it also facilitates mutant p53 degradation is a pertinent question, as it would suggest the existence of a regulatory loop. Another relevant activity of mutant p53 in conjunction with NRF2 is the ability to upregulate antioxidant thioredoxin. Notably this is a gene expressed at high basal levels (Lisek et al., 2018). It is also relevant to add at this point that ROS in cancer cells is actively suppressed by a number of other oncogenes (e.g., Myc, K-Ras, B-Raf) which increase NRF2 transcription, and in turn upregulate vital antioxidant pathways (DeNicola et al., 2011).

In contrast however, mutant p53 is also identified to increase ROS, in male-dominated oesophageal cancers and also lung cells. This is driven through its NRF2-mediated inhibition of SLC7A11 (glutamate/cystine antiporter system), which in turn depletes glutathione. This has scope for therapy [as demonstrated by our collaborators with us (Liu et al., 2017)] and given the context, is predicted to have particular relevance to males. This is compounded by mutant p53 and NRF2 suppression of heme oxygenase I, a gene generally at low basal levels (Lisek et al., 2018).

To understand this apparent dichotomy, it is important to be aware that ROS has distinct impact depending on the stage of cancer development. Cancer initiation is primed by chronic exposure to modest ROS levels, whereas acute high levels have greater chance of inducing cell death. At an advanced stage in transformation however, ROS is necessary for promoting a highly metastatic phenotype (Lisek et al., 2018 and references within). This appears an oversimplified version of understanding even so, as antioxidant treatment of cancers can in some instances promote rather than inhibit.

The capacity of cells to manipulate ROS levels according to their situation requires diversity in regulatory ability. Discovery of both modes of control, although initially puzzling, is being heavily studied [e.g., where antioxidants can increase metastasis in melanomas; which is a disease predominate in males (Le Gal et al., 2015)]. It is relevant to determine how these disparate outcomes of mutant p53-NRF2 engagement reflect the cell type and their sexes, in addition to cancer stage.

## Conclusion

P53 is intimately linked with cancer sex-disparity. A critical role of p53 activity, finely balancing cancer prevention and aging is evident. Inherited polymorphisms in p53 pathways are noted for their influence in select ethnic populations, with some evidence of sex-disparities that lack a clear universal trend. These findings predict that historically, advantage was gained from these SNPs under particular conditions, causing their biased selection. It is likely that in the future, the expanding banks of human DNA sequence data will offer fresh insight into the relevance of these polymorphisms to male and female survival. Greater female longevity among individuals with weakened p53, who avoid cancer death (“Altered TP53 Regulation and Its Cancer Risks for Females and Males” section), remains to be interrogated with the promise of therapeutic relevance.

Uncontrolled free radicals are a major DNA mutation risk, with overall greater danger in males for many reasons. Among these gene mutations, *TP53* is outstanding in prevalence and a disproportionate burden in males with non-reproductive cancer. This is a danger not simply for the loss of wt p53 anti-cancer defence capacity, but additionally for the acquisition of new oncogenic capacities (“Cancer Sex Disparity Linked to Redox Activity Through p53” section).

This review offers a very preliminary glance into the breadth of factors that may influence p53 function in different ways in males and females. These findings caution against attempting to simplify understanding by looking only at single parameters out of context. This is definitely a challenging approach to understanding cancer, but at the dawn of sex and gender medicine, it is extremely timely to raise the importance of these considerations for cancer.

## Author Contributions

SH researched and wrote this review and drew the associated figure. YH contributed to the writing and reviewing of the manuscript. Both authors contributed to the article and approved the submitted version.

## Conflict of Interest

The authors declare that the research was conducted in the absence of any commercial or financial relationships that could be construed as a potential conflict of interest.
